# Ectopic Thymic Tissue Presenting as an Epiglottic Mass Compromising a Neonatal Airway: A Case Report

**DOI:** 10.7759/cureus.75164

**Published:** 2024-12-05

**Authors:** Abraham Oommen, Tyler Gross, Catherine Preedy, Elizabeth O'Donnell, Nicole L Aaronson

**Affiliations:** 1 Department of Anesthesiology and Perioperative Medicine, Nemours Children's Health System, Wilmington, USA; 2 Department of Anesthesiology and Perioperative Medicine, Thomas Jefferson University Hospital, Philadelphia, USA; 3 Division of Neonatology, Department of Pediatrics, Nemours Children's Health System, Wilmington, USA; 4 Division of Otolaryngology, Department of Surgery, Nemours Children's Health System, Wilmington, USA

**Keywords:** aberrant thymic tissue, airway mass, anesthetic challenge in neonate, epiglottic cyst, pediatric case report, upper airway compromise

## Abstract

An epiglottic mass (EM) is rarely found in neonates and poses life-threatening airway complications. We present the case of an infant urgently transferred from Belize via the World Pediatric Project with a lingual EM. The EM was misdiagnosed twice. The patient's home country of Belize initially misdiagnosed the EM as an elongated uvula and then again as an esophageal polyp. The true nature of the upper airway mass (UAM) was only discovered intraoperatively on rigid bronchoscopy as a lingual, mobile EM. Pathology of the lingual EM showed fibrovascular tissue with an area of exuberant capillary proliferation, cartilage, and ectopic thymic tissue. A second, smaller mass was also discovered in the left piriform sinus. The pathology revealed the presence of fibrovascular tissue, mucus glands, and a small amount of skeletal muscle. To the best of our knowledge, a thorough review of the literature reveals that this is the first description of ectopic thymic tissue presenting as an EM.

## Introduction

The presentation of epiglottic masses (EMs) in neonates and infants can be varied and may include stridor, choking, gagging, feeding difficulty, poor weight gain, airway obstruction, and death in more serious cases [1-3]. Although rare, EMs are typically solitary and may be cystic, granulomatous, or neoplastic [4,5]. Regardless of etiology, evaluation and removal of symptomatic masses is especially important in infants due to their smaller airway anatomy and risk of mechanical asphyxiation with larger, mobile masses [6]. A recent report by Arteaga et al. describes the utility of ex-utero intrapartum treatment (EXIT) of upper airway masses (UAM) diagnosed antenatally in preventing hypoxic brain injury and controlled tracheal intubation [7]. Cases of UAM are often complex, requiring multiple attempts at visualization before securing the airway for operative procedures [7-9].

The differential diagnosis of UAM includes vallecular cysts (VC), cystic hygroma, teratoma, hamartoma, lymphangioma, thyroglossal duct cyst, and thyroid remnant cyst [10,11]. Clinically significant UAMs in neonates and infants commonly produce a mass effect that necessitates surgical intervention. If the UAM is large enough, dynamic changes can obstruct the view of the glottis and cause life-threatening respiratory compromise [6]. An EM is rare, and two symptomatic UAMs of differing pathologies have not been reported previously. Our case is the first to our knowledge of ectopic thymic tissue presenting as a pedunculated EM requiring urgent operative management. Written Health Insurance Portability and Accountability Act (HIPAA) authorization has been obtained from the patient’s mother to publish this case report. This case was presented at the American Society of Anesthesiologists annual meeting on October 19, 2024, in Philadelphia.

## Case presentation

A 36-week gestational-age female infant was delivered in Belize via cesarean section due to premature prolonged rupture of membranes. She had a birth weight of 2381 g. Shortly after birth, she exhibited nasal flaring and signs of mild respiratory distress, necessitating supplemental oxygen through a nasal cannula. She experienced some respiratory distress during both breast and bottle feeding, though her condition was gradually improving. Upon discharge, her physical examination was largely normal, except for an oropharyngeal evaluation that revealed an "elongated uvula." There was no immediate access to an otolaryngologist at the hospital where she was born.

**Figure 1:**
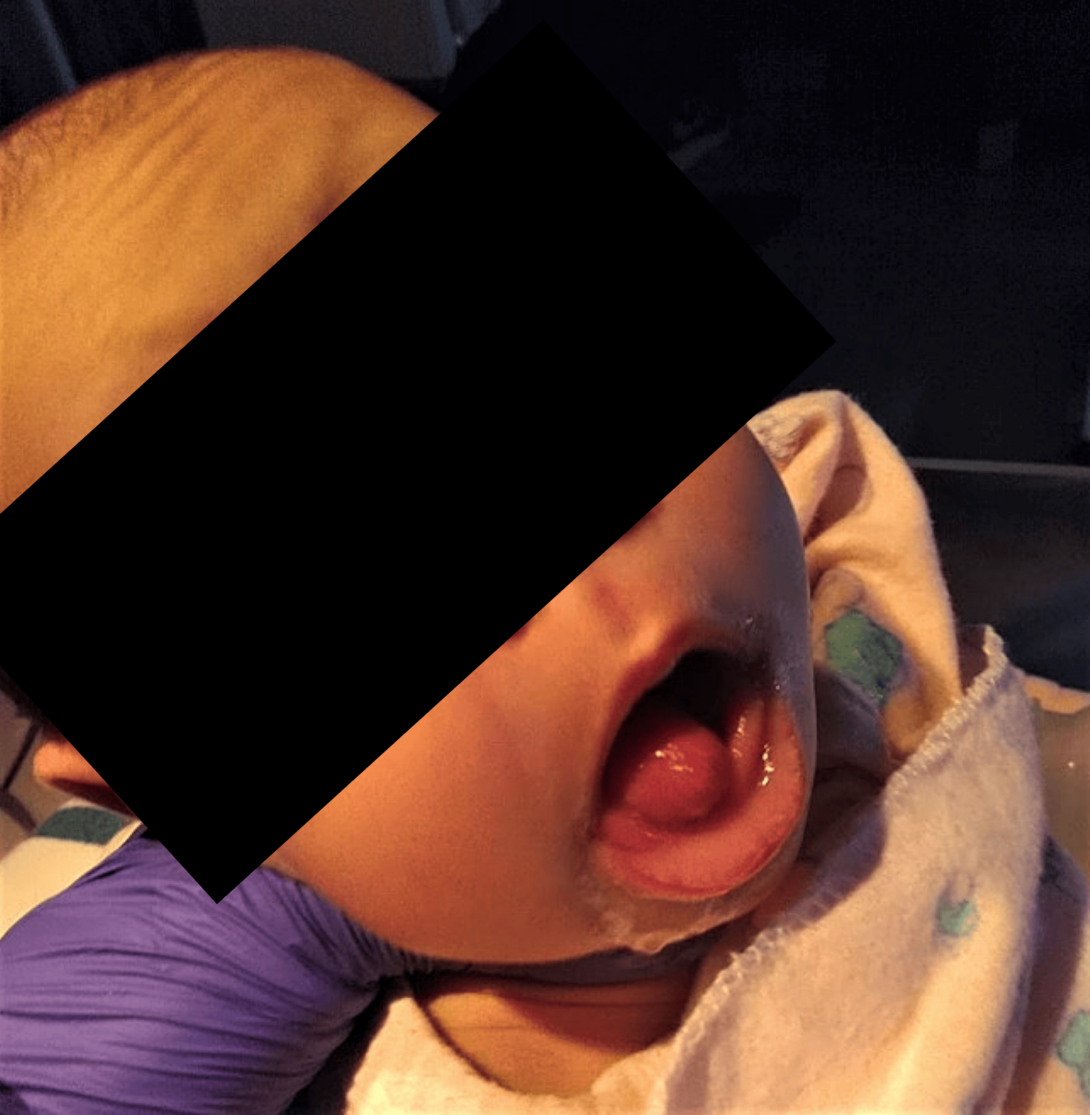
Epiglottal mass protruding from the oropharynx initially considered elongated uvula vs. esophageal polyp.

On the seventh day of life, the mother brought her infant to the emergency department of a local hospital, expressing concern that the baby had spit up during breastfeeding and that she had observed a mass protruding from the neonate’s mouth (Figure 1). By the 17th day of life, the baby was diagnosed with an esophageal diverticulum. Subsequent imaging performed in Belize revealed an esophageal polyp measuring 40 mm × 90 mm.

A U.S. pediatric surgery team assessed the infant during their visit. Unfortunately, surgical intervention was not feasible due to a lack of resources in the patient’s home country. At 11 weeks of age, the infant was internationally transferred to our facility. Upon admission, she weighed 2750 g.

A challenging airway was anticipated due to the patient's condition. Our primary concern was how the mass would respond under general anesthesia (GA), particularly with the risk of mechanical asphyxiation. Given the signs of respiratory compromise and the patient’s ongoing weight loss, the otolaryngology team decided to proceed urgently with operative exploration. Although the possibility of utilizing extracorporeal membrane oxygenation was considered, it was ultimately determined that, in the event of mechanical asphyxiation, an immediate tracheostomy would be a more effective intervention.

GA was induced while maintaining spontaneous ventilation, anticipating potential challenges with airway patency. A gradual combined intravenous and inhalational induction technique was utilized, administering 2 mg/kg of propofol with 4% inhaled sevoflurane in oxygen until a minimum alveolar concentration (MAC) of 1 was attained. Mild upper airway obstruction was noted, which improved with the application of continuous positive airway pressure (CPAP) of 5 mm Hg. Once the infant reached an adequate depth of GA, we were able to assist with ventilation without any complications.

The otolaryngology team attempted to visualize the larynx using direct laryngoscopy, but the procedure was challenging due to the distorted upper airway anatomy. Three attempts at direct laryngoscopy were unsuccessful using Miller 0 and Miller 1 blades. We then opted for indirect video laryngoscopy using the C-MAC system (Karl Storz, Tuttlingen, Germany), which clearly displayed the distorted upper airway anatomy. It became clear that the mass was a UAM and not an esophageal polyp as previously described by physicians from the patient's home country. The larynx was deviated towards the side of the EM. After two attempts with indirect video laryngoscopy, we were able to secure the airway with a 3.0 cuffed endotracheal tube (ETT) using the C-MAC Macintosh 0 blade. The ETT cuff was inflated with 0.5 ml of air and taped at 10 cm at the gum. The time from induction of anesthesia to successful intubation was 22 minutes. GA was maintained using a combination of inhaled sevoflurane at an MAC of 1 and 4 mg of rocuronium for muscle relaxation. Once the airway was secured, we opted to use muscle relaxation to optimize surgical conditions with planned extubation in the neonatal ICU on postoperative day 1.

**Figure 2:**
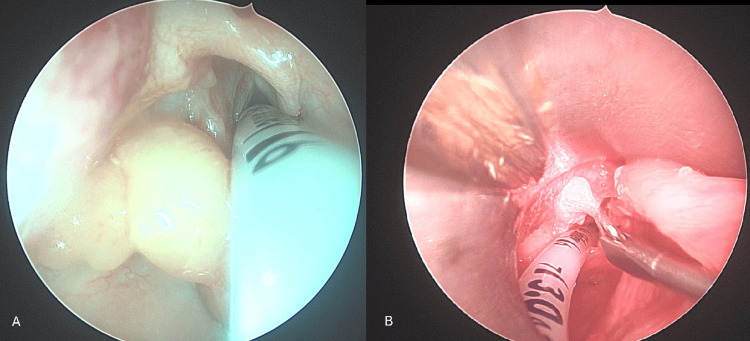
Intraoperative images showing left globular piriform mass (A) and lingual epiglottic mass (B). Note the surgical resection underway at the base of the stalk in image B.​

**Figure 3:**
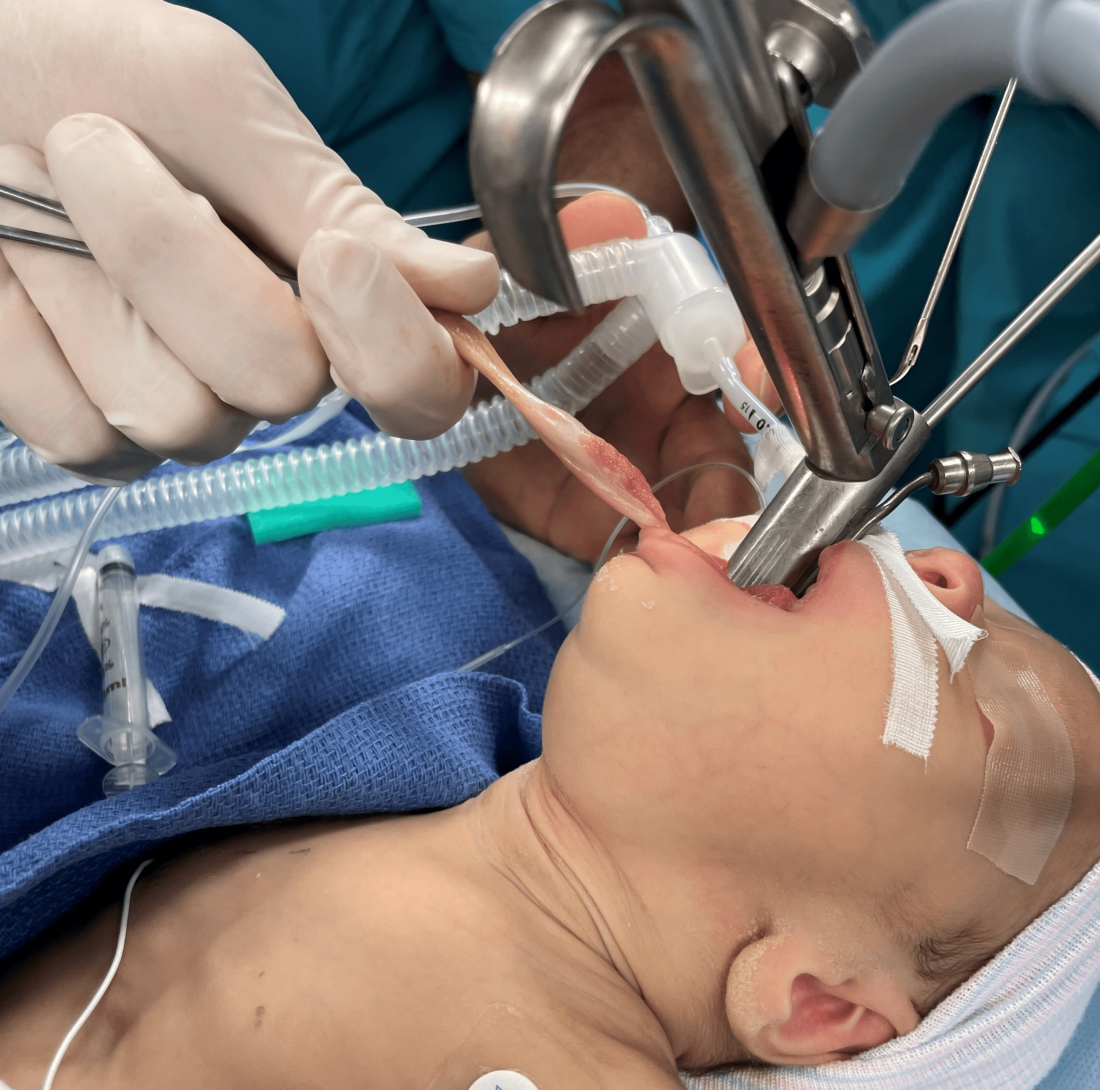
Surgical traction on the lingual epiglottic mass to expose the base of the mass for resection.

**Figure 4:**
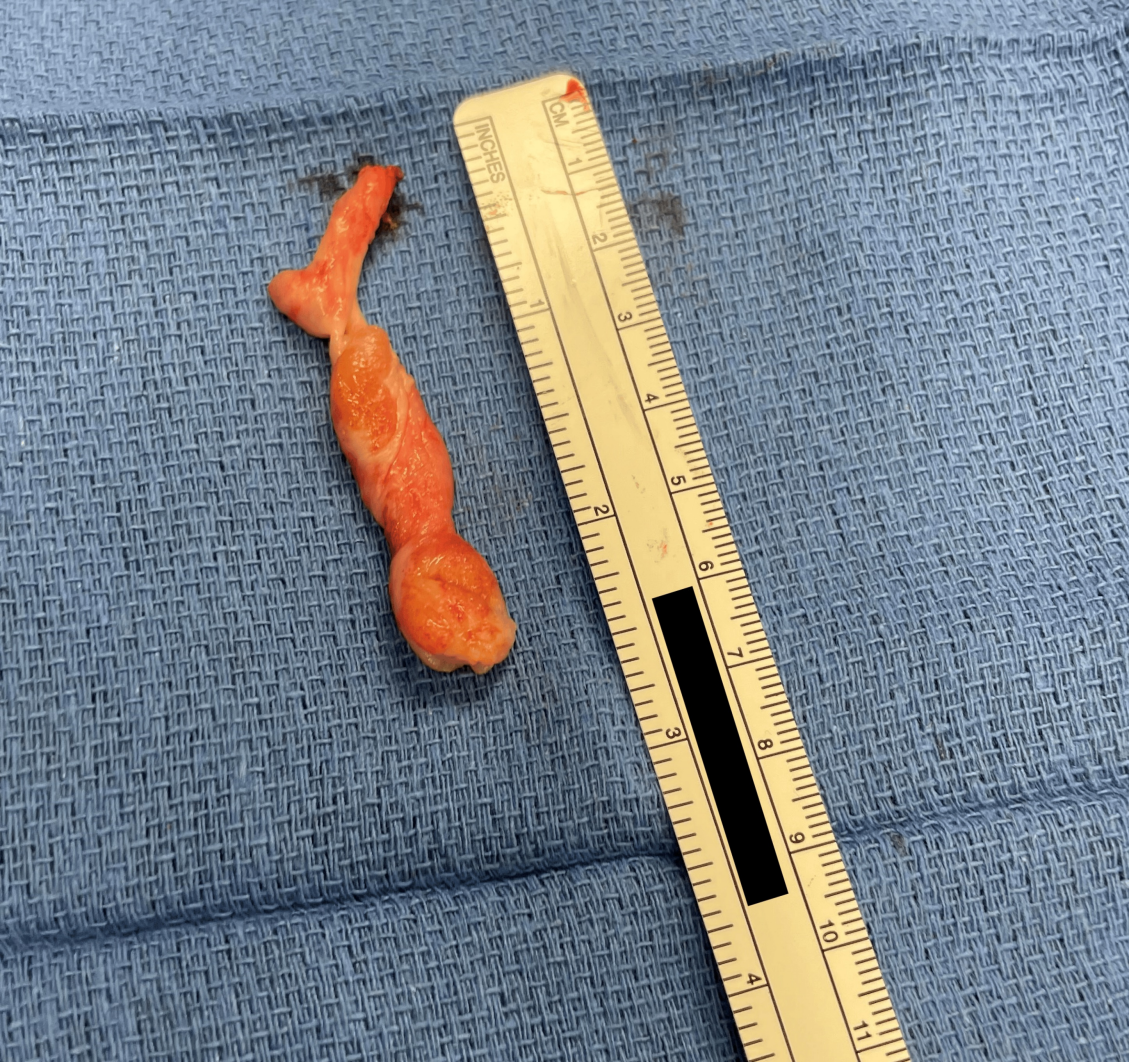
Resected mass measuring just over 6.5 cm.

**Figure 5:**
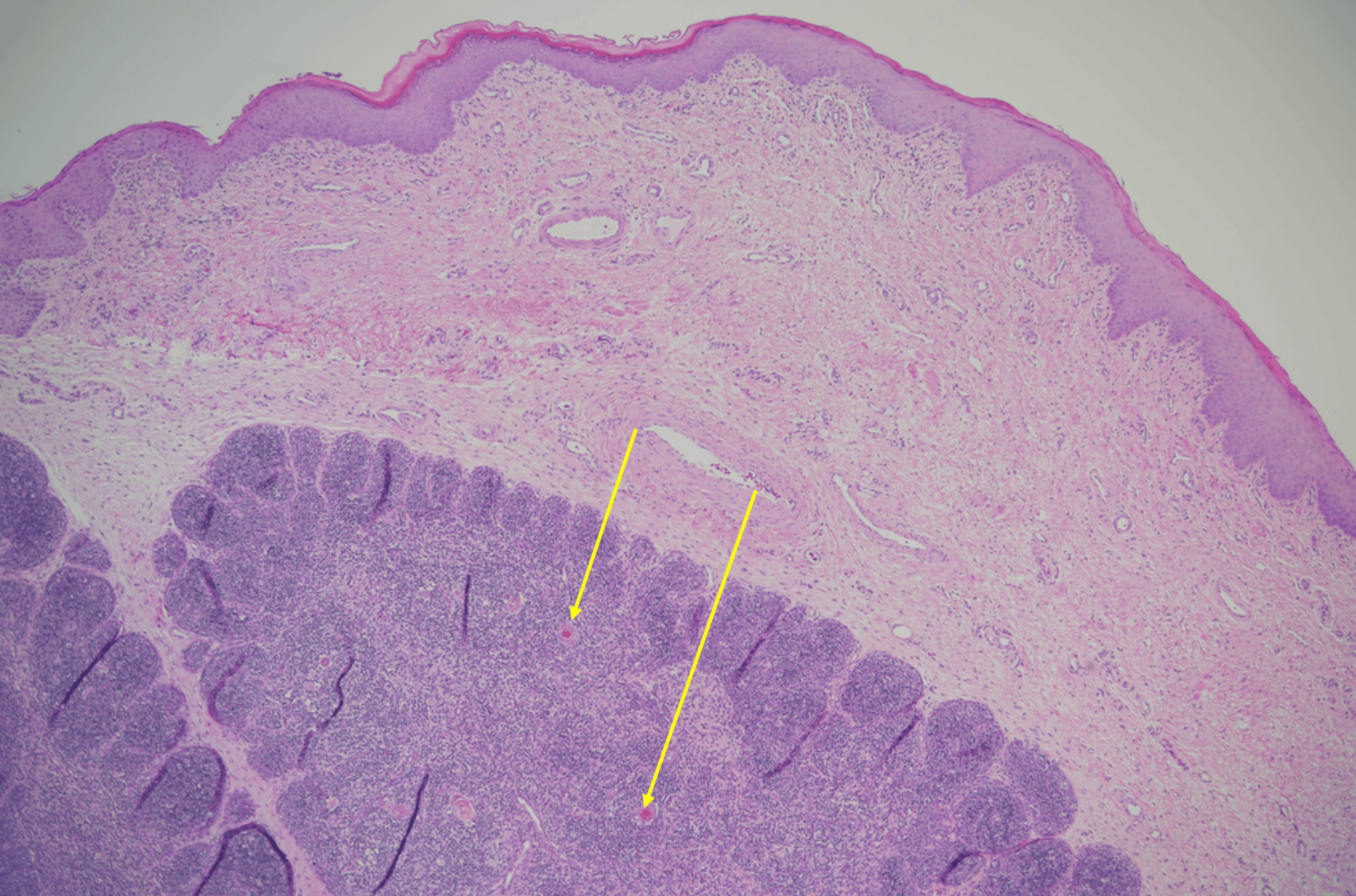
Histopathology showing evidence of Hassall's corpuscles, also known as thymic bodies (indicated by yellow arrows).

Rigid bronchoscopy revealed two distinct masses (Figure 2). There was a left piriform mass and a right-sided lingual EM. The lingual EM required external traction to expose the base of the mass for resection (Figure 3). The resected mass is seen in Figure 4. The pathology report for the lingual EM showed a polyp measuring 6.5 cm × 1.5 cm composed of fibrovascular tissue, exuberant capillary proliferation, cartilage, and ectopic thymic tissue covered by squamous epithelium (Figure 5). The left piriform mass showed a polyp measuring 1.1 cm × 0.4 cm × 0.3 cm composed of fibrovascular tissue, mucus glands, cartilage, and a small focus of skeletal muscle covered by squamous epithelium.

At the end of the procedure, the infant was transferred to the neonatal ICU, where she was extubated without complication on postoperative day 1. The infant began to gain weight appropriately through a combination of breast and bottle feeding. An evaluation by our speech and language team indicated there were no further concerns regarding her feeding. Her respiratory symptoms improved significantly, and her stridor resolved by the time of discharge. After a 15-day hospital stay, she was discharged for outpatient follow-up. Mother and baby returned to Belize in excellent condition two weeks after discharge.

## Discussion

Since 1987, there have been seven case reports involving ectopic thymic tissue in the subglottic region of neonates and infants resulting in respiratory distress and stridor [12]. There is one case report of periglottic ectopic thymic tissue found on autopsy, which resulted in perinatal asphyxia after extreme difficulty with intubation, but no reports of ectopic thymic tissue resulting in an EM or UAM [8]. This is the largest reported airway mass composed of ectopic thymic tissue. This mass is also large in the context of UAMs of other etiologies, as one study describing VCs included eight cases with a mean cyst size of 8 mm (range, 4-12 mm) [1]. The EM in our patient measured 6.5 cm in length, which is eight times larger in size when compared to the mean VC.

Our knowledge of airway masses in fetuses and neonates continues to grow. These airways present some of the most challenging scenarios. EXIT procedures have been described as a safe transition from intrauterine to extrauterine life, where successful intubation while on placental support precedes final delivery [7,13]. Given Belize's medical and healthcare limitations, our patient had no option for an EXIT procedure.

The anesthetic and airway management of a patient with one UAM is complex. Managing two discreet airway masses was an even greater challenge. The size, mobility, and propensity for dynamic obstruction are the most concerning variables that are difficult to predict. EMs in neonates and infants have a wide range of pathological presentations, with most patients developing symptoms within the first few weeks of life [2]. To definitively diagnose a UAM, direct visualization followed by resection and histological examination are required [5]. We agree with Artega et al. that video laryngoscopy should be used to initially secure the airway [7]. Considering the difficulty in identifying the airway once the infant was anesthetized, visualization while mildly sedated but spontaneously breathing may have been a more prudent choice. Definitive treatment of the mass via complete resection resulted in decreased stridor, improved feeding tolerance, weight gain, and overall greater quality of life for our patient. Anesthetic care of neonates with UAMs may lead to critical scenarios secondary to altered airway anatomy, respiratory distress, and resultant hypoxic injury. Cautious planning is paramount, and intubation via an EXIT procedure in a controlled setting might become the standard of care in UAM cases diagnosed antenatally.

## Conclusions

A neonatal airway mass is a rare finding but is a potentially life-threatening condition that requires prompt diagnosis and management. These masses arise from various etiologies, including congenital anomalies such as cystic hygromas, teratomas, or vascular malformations. It is our aim that clinicians will consider ectopic thymic tissue as part of the differential diagnosis when treating UAMs. Multidisciplinary collaboration, often involving anesthesiologists, pediatric otolaryngologists, and neonatologists, is essential for planning appropriate interventions. In some cases, securing the airway through intubation or tracheostomy may be required to stabilize the neonate before definitive treatment. Early recognition and intervention are vital to prevent complications and ensure the best outcome for affected neonates.
